# Site-directed *in vitro *immunization leads to a complete human monoclonal IgG4λ that binds specifically to the CDR2 region of CTLA-4 (CD152) without interfering the engagement of natural ligands

**DOI:** 10.1186/1472-6750-7-51

**Published:** 2007-08-23

**Authors:** Li-Te Chin, Chishih Chu, Han-Min Chen, Shu-Ching Hsu, Bor-Chun Weng, Chi-Hong Chu

**Affiliations:** 1 Graduate Institute of Life Science, Fu-Jen Catholic University, Taipei, Taiwan, RoC; 2 HumOrigin Biotechnology Corp., Hsinchu, Taiwan, RoC; 3 Graduate Institute of Medical Sciences, National Defense Medical Center, Taipei, Taiwan, RoC; 4 Department of Microbiology and Immunology, National Chiayi University, Chiayi City, Taiwan, RoC; 5 Section of General Surgery, Department of Surgery, Tri-Service General Hospital, National Defense Medical Center, Taipei, Taiwan, RoC

## Abstract

**Background:**

The ability to acquire fully human monoclonal antibodies (mAbs) with pre-defined specificities is critical to the development of molecular tags for the analysis of receptor function in addition to promising immunotherapeutics. Yet most of the arriving affinity maturated and complete human immunoglobulin G (IgG) molecules, which are actually derived from single human B cells, have not widely been used to study the conserved self antigens (Ags) such as CD152 (cytotoxic T lymphocyte antigen-4, CTLA-4) because proper hosts are lacking.

**Results:**

Here we developed an optimized protocol for site-directed *in vitro *immunizing peripheral blood mononuclear cells (PBMC) by using a selected epitope of human CD152, an essential receptor involved in down-regulation of T cell activation. The resultant stable trioma cell lines constantly produce anti-CD152 mAb (γ4λhuCD152), which contains variable (V) regions of the heavy chain and the light chain derived from the VH3 and Vλ human germline genes, respectively, and yet displays an unusual IgG4 isotype. Interestingly, γ4λhuCD152 has a basic pI not commonly found in myeloid monoclonal IgG4λs as revealed by the isoelectric focusing (IEF) analysis. Furthermore, γ4λhuCD152 binds specifically, with nanomolar affinity, to an extracellular constituency encompassing the putative second complementarity determining region (CDR2) of CD152, whereby it can react to activated CD3^+ ^cells.

**Conclusion:**

In a context of specific cell depletion and conditioned medium,*in vitro *induction of human Abs against a conserved self Ag was successfully acquired and a relatively basic mAb, γ4λhuCD152, with high affinity to CDR2 of CD152 was thus obtained. Application of such a human IgG4λ mAb with designated CDR2 specificity may impact upon and prefer for CD152 labeling both *in situ *and *ex situ*, as it does not affect the binding of endogenous B7 ligands and can localize into the confined immunological synapse which may otherwise prevent the access of whole IgG1 molecules.

## Background

Fueled by ever-growing demand, complete human mAbs have become one of the most important disciplines for obtaining research and therapeutic leads. Currently, the identification of such materials with desired specificities requires either selecting from artificial genetic Ig libraries [1,2] or immunizing transgenic mice that harbored large human Ig loci [3,4]. Unfortunately, because of their dependence on Ig gene shuffling, information about the original pairing of heavy (H) and light (L) chains inherent in a single human B cell has been limited. An alternative strategy for obtaining complete human mAbs would be to use combined heterotopic B- and T-cell epitopes as an immunogen in human lymphocyte cultures, followed by standard hybridoma and/or cloning procedures. Initially, the validity of this site-directed *in vitro *immunization approach has been established in the procurement of gp120-specific monoclonal IgM from seronegative, non-infected lymphocytes [5]. Viral neutralizing, affinity maturated and isotype switched IgG responses were subsequently confirmed in human naïve B lymphocytes [6-8]. However, from prior reports, it was unclear whether B-cell epitopes present on a self-protein would also elicit significant IgG responses in the site-directed *in vitro *immunization regimen; therefore, a molecule with its existence on lymphocytes represents an ideal candidate for such a study.

CD152 belongs to a group of immunomodulating receptors, collectively termed as CD28 superfamily [9], and represents one of the major inhibitory receptors involved in co-stimulatory pathways regulating both humoral and cellular immune response [10,11]. These inhibitory effects are due in part to a higher avidity of binding by the common endogenous agonists, B7-1 (CD80) and B7-2 (CD86), compared with its stimulatory homologue, CD28 [12,13]. The lurch toward CD152 of these agonists reduces T-cell proliferation and cytokine production, resulting in attenuated immune responses, and thus mediates tolerance and/or anergy [14,15]. CD152 has also been demonstrated to promote clonal anergy development by limiting cell cycle progression during the primary response *in vivo *[16], thus CD152 opened up the possibility to study whether the current knowledge in site-directed *in vitro *immunization allows any generalizations to be made that will consequently be useful in developing human mAbs against self Ags.

Structural findings indicate that the CD152 protein is composed of disulfide-linked homodimers of extracellular IgV domains. Each domain consists of two layered β-sheets with ten strands (A, A', B, C, C', C", D, E, F and G) [17-19]. Furthermore, one mutational [20] and two crystallographic [17,18] studies have independently pointed out that CDR1-like (the B-C loop) and CDR3-like (the F-G loop) regions in CD152 directly bind B7 ligands, whereas the role of CDR2 was very insignificant, if it played a part at all. In contrast to the harmonized results to the relative contribution of individual CDR's, a severe discrepancy existed even in the span of CDR2. In the mutational model, the extracellular consecutive ^51^AATYM^55 ^motif was implicated to be CDR2 [20] whereas co-crystallographic structures characterized the C'-C" loop encompassing a single Met 55 as CDR2 [17,19]. To further complicate the picture of functionality, the downstream M10 (^59^ELT^61^) and M11 (^66^SICT^69^) epitopes, localized between the C" and D strands, have been revealed to play an important pharmacological role upon Ab binding [21]. Thus not only is the dimension of CDR2 controversial but also, additional domains potentially involved in certain functions of CD152 are suggested. The suggestion of an important contribution in this area defined by the Met 55 core (^51^AATYMMGNELTFLDDSICT^69^) is further strengthened by observing a considerable conservation across all identified CD152, with six of the 19 amino acids having identical residues to the human sequence [19].

Here we explored the stimulating effect of the Met 55-cored sequence by invoking the optimized process of site-directed *in vitro *immunization and somatic cell hybridization [22] to target this particular area. We showed that human Abs that are specific for the CDR2-encompassed Met 55-cored region could be regularly generated *in vitro *from normal donors after sensitization with a heterotopic peptide. Moreover, the resultant human IgG4λ mAb is useful to probe and label CDR152 *in situ *and *ex situ *during the responses involving such a particular self molecule.

## Results

### Procuring the desired Ab response by site-directed *in vitro *immunization and electrofusion

**Figure 1:**
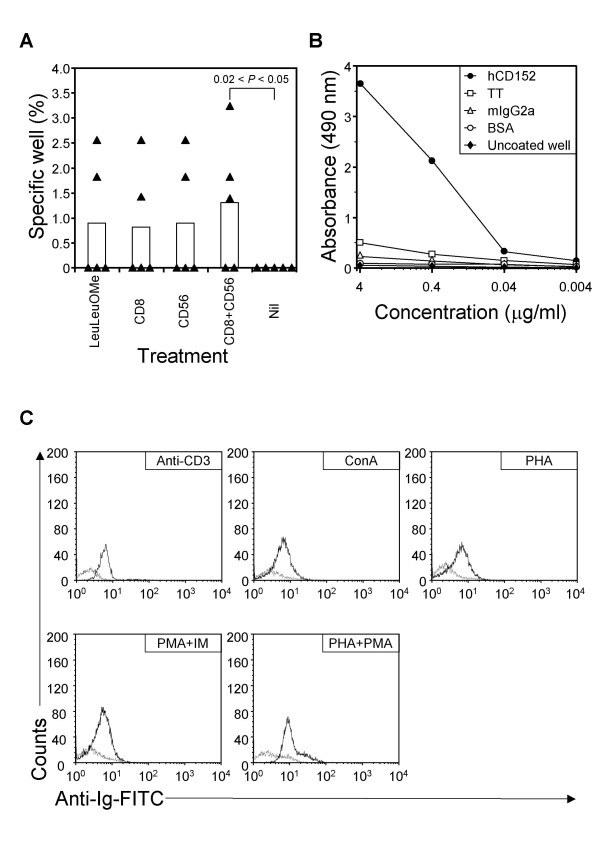
**Anti-CD152 derived from site-directed *in vitro *immunization exhibits specific binding towards human CD152 expressed both as a recombinant protein as well as a cell surface receptor**. Panel A summarizes specific responses of PBMC's from five subjects receiving primary Ag stimulation alone (Nil) or from PBMC's treated with the indicated regimen. Bars indicate the median value of all subjects analyzed. Filled triangles represent specific frequencies of individual PBMC's. Original data can be retrieved from Additional File 1. Panel B illustrates a representative ELISA reactivity profile of culture supernatant. Diluted supernatants were tested in duplicate with 100 μL added to each well. Deviation between duplicate was less than 10% for any reported value. Panel C represents immunofluorescent staining of mitogen-activated CD3^+ ^T cells (right thick peaks). PBMCs were isolated from normal blood donors and stimulated with indicated mitogens for 72 h to induce CD152 expression. Expression of mAb-recognized epitope was verified in gated CD3 population by flow cytometry. Data represent one experiment from three different normal subjects. Appropriate isotype controls were used for all Abs. The basal fluorescence obtained with the use of isotype control (IgG4λ myeloma protein) is indicated by the left thin peaks.

Plasma samples from repeated healthy donors were initially screened for IgG and IgM against tetanus toxoid, rhCD152 and mouse IgG2a by ELISA. It was found that no donors (0/17) had any significant IgM or IgG responses to CD152; however, anti-tetanus toxoid IgG (15/17) and some, albeit low, levels of specific IgG antibody against mouse IgG2a (2/17) were found. After primary and secondary *in vitro *immunization of five PBMC's from individual donors, the respective EBV-activated lymphoblastoid cultures were screened for human Igs against CD152. When assayed by ELISA, frequencies of specific cultures detected in the lymphoblastoid cells after primary *in vitro *immunization ranged from 0% to above 3% and were significantly elevated when CD8^+ ^and CD56^+^cells were simultaneously removed before primary peptide stimulation (Fig. 1A).

As shown in Additional File 1, although there was not a statistically significant increase, the removal of IL-10^+ ^cells before secondary peptide stimulation yielded the highest frequency of specific IgG-producing cells.

Wells containing anti-CD152 IgG level that was five times higher than anti-murine IgG2a were subsequently pooled for electrofusion. One fusion yielded eleven clones secreting anti-CD152 Abs. Three of them were subcloned and found to be of the IgG4 isotype. To select a mAb for further characterization, two parameters were taken into account: the secretory capacity of the trioma, estimated from the ELISA titer and the relative specificity, reflected by the reactivities to other unrelated antigens such as tetanus toxoid, bovine serum albumin, and uncoated wells. Figure 1B also demonstrates that the selected monoclonal had a near-background reaction with unrelated Ags. Long-term (over a period of 60 months of continuous culture) stable Ab production in a concentration above 4 μg/ml/10^7 ^trioma cells in spent medium was consistently observed.

### Presentation of recognized epitope on mitogen-activated human T cells and the immunogen

To investigate whether the specificity observed in ELISA was also applicable for the activated human T cells *in situ*, we used the mitogen-dependent stimulation system, where peripheral T cells were activated with anti-CD3, PHA, ConA, or the combination of PMA/ionomycin or PHA/PMA. CD152-expressing T cells were numerated in CD3^+ ^population by flow cytometry. After a 72-h culture period, few CD3^+ ^T cells without stimulation were stained by our human mAb whilst a large number of CD152^+^CD3^+ ^T cells were constantly observed after activation with PMA and PHA (Fig. 1B). Control human IgG4λ myeloma protein failed to distinguish among the activation statuses and the present mAb did not react with activated murine CD3^+ ^T cells (data not shown).

**Figure 2:**
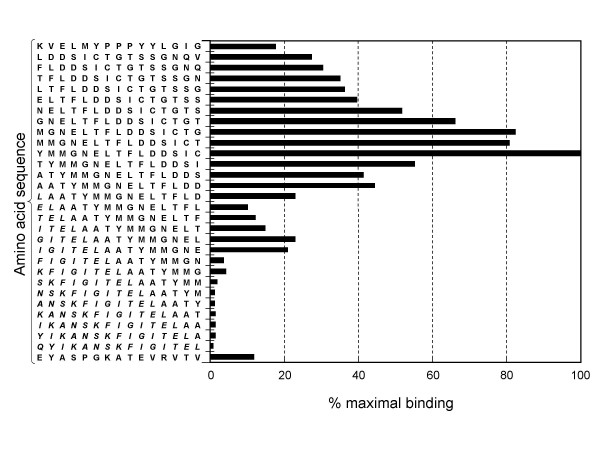
**Immunoblot screening of peptide arrays denotes the mAb specificity against the Met 55-cored CDR2-like region of human CD152**. 1 μg/mL of purified mAb was used to determine the binding epitope. Sequences of EYASPGKATEVRVTV, KVELMYPPPYYLGIG and QYIKANSKFIGITEL indicate CDR1-encompassing region, CDR3-encompassing region and T cell epitope (italic) used for site-directed immunization, respectively.

To further characterize the nature of mAb binding, epitope mapping was performed by the Western blotting method with arrays containing the overlapping pendecapeptides, encompassing CDR1-like (the B-C loop), CDR3-like (the F-G loop) and the Met 55-cored sequence localized between the C' and D strands of the CD152 extracellular portion. Figure 2 depicts that only the peptide corresponding to the C-terminus of the Met 55-cored sequence (^54^YMMGNELTFLDDSIC^68^) was best recognized by the mAb while neither the promiscuous T-cell epitope, nor CDR1-like or CDR3-like region contributes to the binding. From this result, it can be concluded that the Ala 51, Ala 52, Thr 53 and Thr 69 are not essential for mAb recognition.

### Immunological and physicochemical properties of the mAb

**Figure 3:**
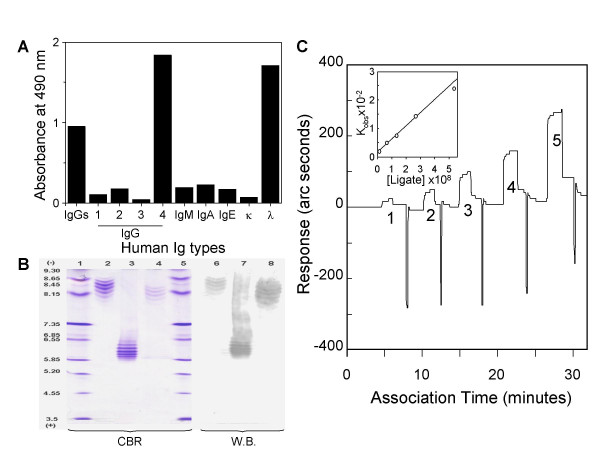
**Immunological and biochemical natures of the mAb**. Panel A shows isotyping and subtyping results by immobilizing anti-human Igs as indicated. The binding profile of the mAb was subsequently revealed by biotinylated CD152-muIg and avidin-peroxidase conjugates. Results indicate that the present mAb belongs to a type of IgG4λ. Panel B indicates the isoelectric point of the monoclonal IgG4λ anti-CD152 (lane 4 and 8), resolved by isoelectric focusing. Monoclonal human myeloma IgG1λ(lane 2 and 6) and IgG4λ (lane 3 and 7) were run in parallel for comparison. The electrophoretic patterns were visualized by either Coomassie brilliant blue staining (lane 1–5) or immunoblot (lane 6–8) with anti-human IgG conjugated with peroxidase and FAST™ DAB. The calculated isoelectric points for human IgG1λ, IgG4λ and anti-CD152 mAb to be approximately in the range of 7.92–8.79, 5.76–6.52 and 7.87–8.41, respectively, based on the calibration against the linear regression of standard protein markers. Panel C outlines the affinity determination by IAsys. Surface plasmon resonance obtained at 25°C for increasing concentrations of anti-CD152 mAb on purified, unlabeled CD152-muIg. The straight line in the inset was obtained from the k_obs _plot versus ligated Ab concentration and yielded a k_diss _(the intercept) of 16.81 and a k_ass _value (the gradient) of 4.20 × 10^9^. Therefore produced a Kd (k_diss_/k_ass_) of 4 × 10^-9 ^M.

The mAb was isotyped and subtyped by solid phase ELISA, utilizing its reactivity with human CD152 and appropriate immobilized typing Abs. The binding depicts the simultaneous presence of γ4 and λ chains in the mAb while other Ig chains are absent (Fig. 3A), thus the mAb was termed as γ4λhuCD152. Additionally, to compare the clonal nature with existing human monoclonal IgG1λ and IgG4λ derived from purified myeloma proteins, the isoelectric focusing (IEF) patterns were subsequently visualized. The resolvable bands, as shown in Figure 3B, indicate that γ4λhuCD152 has a slightly lower yet basic pI similar to the IgG1λ, but in contrast to the acidic IgG4λ myeloma protein or to the anionic (pI 4.5–5.0) species of proteins commonly described for polyclonal IgG4 [24]. Western blot confirmed the purity of the Ab samples as illustrated by the anti-λ staining configurations. As shown in Figures 3B, the corresponding pI of the myeloid IgG1λ, IgG4λ and γ4λhuCD152 obtained with linear regression of pH gradient were 7.92–8.79, 5.76–6.52 and 7.87–8.41, respectively.

The equilibrium dissociation constant (Kd) for the purified intact γ4λhuCD152 was determined by an IAsys analysis. The rate constant was evaluated directly from the sensogram using five cycles of soluble mAb binding to the immobilized CD152-muIg. Figure 3D reveals that, with the analysis of extent and association in single phase, the Kd was deduced to be 4 × 10^-9 ^M.

### Ig sequences

**Figure 4:**
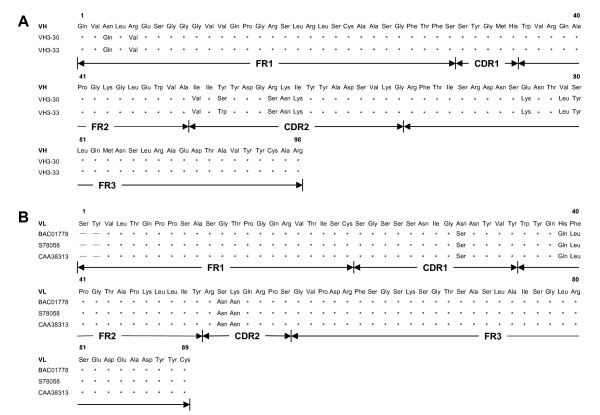
**Partial deduced protein sequences of the mAb**. Panel A represents the alignments of VH to known human VH sequences of the highest homology scoring. Panel B represents the alignments of VL to known human VL sequences of the highest homology scoring. FR, framework region; CDR, complementarity-determining region. Asterisks indicate amino acid identity to germline. Sequences are available from GenBank under accession numbers AY847516 (VH); AY847517 (VL); AB019439 (VH3–30 and VH3–33); BAC01778; S78058 and CAA38313. Homology searches were accomplished over the www using the program BLAST (Basic Local Alignment Search Tool) from NCBI (National Center for Biotechnology Information; Washington, DC.). A 12-aa CDRH3 sequenced Ala-His-Gly-Asp-Tyr-Gly-Arg-Asp-Gly-Met-Asp-Val was noted.

Following elelectrofusion and subsequent clonings, stable trioma cells producing IgG4λ were selected and the cDNA encoding the Ig variable regions were cloned and sequenced. By comparing the VH with the available Ig sequences, it was concluded that the VH, being 89.80% (88/98) amino acid identity (Fig. 4A), is associated with three human VH3 germline segments [GenBank: AB019439, VH3–30 and VH3–33]. Alignments have also disclosed homology of VL to three existing human Vλ germline genes [GenBank: BAC01778, S78058 and CAA38313] with a measure of 92.13% similarity (Fig. 4B). High similarity to accessible V germline genes of human but not other origins is indicative of complete human Ab. Moreover, both VH (CDR2) and VL (CDR1 and CDR2) regions contained evidence of hyper mutations away from the germline.

### Little or no competition to B7- CD152 binding of γ4λhuCD152 both *ex situ *and *in situ*

**Figure 5:**
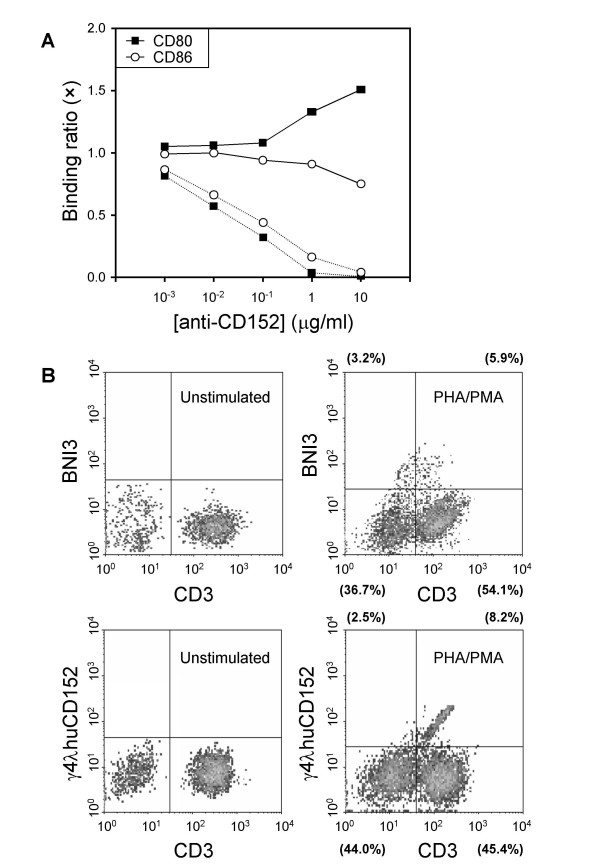
**Binding of γ4λhuCD152 and CD80/CD86 agonists to human CD152 are not mutually exclusive**. Panel A indicates the ligand competition assays that test the ability of monoclonal γ4λhuCD152 (solid line) and BNI3 (dashed line) anti-CD152 to compete for the CD80/CD86 and CD152 interactions. Biotinylated CD80-muIg or CD86-muIg plus indicated increasing concentrations of the mAbs (10^-3^-10 μg/mL) were incubated in microtiter wells coated with purified CD152-muIg. Bound CD80/CD86 was detected with avidin-peroxidase conjugate and a peroxidase substrate. The data shown are representative of three experiments. Panel B illustrates the determination of *in situ *CD152 expression before and after PHA/PMA stimulation. Paraformaldehyde-fixed cells were first labeled with PE-anti-CD3 and BNI3 or γ4λhuCD152, and then incubated with FITC-conjugated anti-mouse IgG2a or anti-human IgGs, respectively. The percentage of each stained population after stimulation is denoted.

Although the CDR2-containing epitope does not seem to be involved in the binding of endogenous cognate ligands (CD80 and CD86) and mAb engagement would not be antagonistic, the epitope might present an allosteric site for non-competitive inhibition. To investigate this possibility, CTLA-4-muIg was immobilized onto wells and either biotinylated CD80-muIg or CD86-muIg was used as a binding ligand in the presence of γ4λhuCD152 or BNI3, *i.e*., a CD152 antagonistic mouse IgG2a mAb [25]. Figure 5A shows, in contrast with the expected dose-dependent inhibition of specific receptor binding by the antagonistic BNI3, γ4λhuCD152 could not compete binding significantly with either CD80-muIg or CD86-muIg *ex situ*. No clear difference in binding was found when competing CD80-muIg or CD86-muIg was used in the binding of γ4λhuCD152 to CTLA-4-muIg (data not shown). Surprisingly, high doses of γ4λhuCD152 mAb display synergism with the natural ligand CD80 but not CD86 with a consequence up to 50% enrichment of CD80 binding to CTLA-4-muIg.

In order to investigate the binding effects more thoroughly, and to provide further information on their possible *in situ *relevance, it was assessed whether labeling of endogenous CD152, a known rare Ag expressed only on activated T cells, by different mAbs, influences the profile of detection. A well-contrasted cytometric picture of total CD152-binding and a clearly distinguishable concentrated pattern of labeling were evidenced on PHA/PMA-activated T cells by using γ4λhuCD152, indicating a lower nonspecific binding over the antagonistic BNI3 (Fig. 5B). Hence, non-ligand competitive γ4λhuCD152 could be used as a new and refined probe which should be useful for sensitive assay and localization of CD152 both *in situ *and *ex situ*.

## Discussion

Previously published techniques for *in vitro *immunization required pre-treatment of PBMC with chemotoxic agents, such as L-leucyl-L-leucin methyl ester hydrobromide (LeuLeuOMe), in order to prevent the "suppressing populations" dominating the response [23]. Unfortunately, residual cytotoxicity and relative redundancy of LeuLeuOMe interferes with subsequent survival and peptide-driven stimulation and therefore the process requires careful timing for the greatest effectiveness. It was found that when Abs were used as more selective reagents to specific removal of CD56^+ ^and IL-10^+ ^cells, no LeuLeuOMe pretreatment is necessary [see Additional File 1]. Advantages of this improved procedure over the conventional procedure are clearly illustrated in the present study, not only in the reduction in sample manipulation, but also in successfully targeting human Ab responses to a pre-determined epitope without the use of experimental animals or sensitized donors. We believe this first reported human mAb directed against a self physiological receptor also signifies a constructive method for recruiting and unleashing the responses to physiological receptors that may not be recognized by donors' own immune system *in vivo*.

We found that the *ex situ *CD152 binding of CD80 increased with the increasing concentration of γ4λhuCD152 (Fig. 5A). Whether this observation also applied *in situ *is yet to be verified. However, human non-antagonistic anti-CD152 scFv fragments, obtained from a synthetic phage library, were indeed documented to synergize with CD80–CD152 but not CD86–CD152 association [26], although to a less extent as compared with the present study. As described above, the CDR2-like Met 55-cored epitope, ^54^YMMGNELTFLDDSIC^68^, on CD152 may not be directly involved in the binding of CD80 but the F-G (CDR3-like) and B-C (CDR1-like) loops provide direct contacts and additional stabilization to CD80, respectively [18,20]. Thus CD80–CD152 binding enhancement by γ4λhuCD152 (and possibly mAbs specific to this particular CDR2-like site) may be attributed to an extended protrusion of the CDR3-like region that facilitates CD80 engagement, or to a further segmentation that fixes the relative orientation of the binding domains for an additional spacing selectivity. These possibilities are not mutually exclusive.

Despite the fact that the present mAb was derived from healthy human donors, several mouse mAbs with similar, but not identical, binding specificities were previously available in literature. For example, mAbs with agonist- or antagonist-like activities against human CD152 were obtained from mice immunized with the recombinant receptor or mitogen-activated human PBMCs in 1995 [27] and 1999 [25]. In the earlier case, the epitopes responsible for the functional activity of the Abs were finely identified. This analysis documented that the CDR2-adjacent epitope (^60^LTFLDD^65^) and the conformational or CDR3 epitope (^102^PPYYL^106^) are responsible for agonist- and antagonist-like activities, respectively. In addition, using a bispecific tandem single-chain variable fragment recognizing ^59^ELT^61 ^and ^66^SICT^69^, another study has revealed a likely CD152 inverse agonist of Ab nature [21]. Ultimately systematic studies are needed to clarify the pharmacological effects of the present human monoclonal IgG4λ with an epitope of a comparable stride (59~69 vs. 54~68).

A conspicuous feature of the present findings is that it is now possible to screen and construct isotype-switched, high affinity human mAbs with pre-defined specificities than previously possible. Consequently, mAbs are able to mimic ligands action commonly found in small synthetic molecules and to facilitate comprehensive receptor-based drug designs. Another peculiarity, as Figure 3A shows the simultaneous presence of IgG4Fc and Cλ human Ig chains associated with the specificity, is that γ4λhuCD152 can be described as a comparable if not a fundamentally authentic human IgG4. Because IgG4 does not activate the complement cascade and it is much more compact than other IgG's [28], accentuating its advantages for acting in the immunological synapse where a spatial limitation is applied [29]. Although the current example comes from CD152, obviously it can also apply to other biological receptors and their cognate ligands.

## Conclusion

In the present study, we started with an improved, more selective *in vitro *immunization protocol and worked toward fully human mAbs against a representing self Ag, CD152. Considerable progress has thus been made in this area as a result of a novel human IgG4λ mAb against CD152. The application of such a mAb with designated CDR2 specificity may impact upon and favor CD152 detection and/or isolation of human CD4^+^CD25^+^CD152^+ ^regulatory T cells [30]. The present study also opens up the possibility of probing and perhaps controlling T cell activation using highly specific, less immunogenic Ig proteins. Further deciphering the biological functions mediated by γ4λhuCD152 may lead to a greater understanding of the regulation and differentiation of immune responses.

## Methods

### Culture materials Ag and Ab reagents

The culture medium used was RPMI-l640 (HyClone, Logan, UT), supplemented with 1 × non-essential amino acids (Life Technologies, Gaithersburg, MD), 10% fetal bovine serum (FBS; Life Technologies) and 50 μg/ml of gentamycin and kanamycin (Sinton Chemical & Pharmaceutical, Hsinchu, Taiwan). Purified and biotinylated human CD152-murine Ig fusion protein (CD152-muIg), CD80-muIg and CD86-muIg (Ancell, Bayport, MN) were used in Ag-specific and competing enzyme-linked immunosorbent assay (ELISA), together with peroxidase-labeled goat antibodies against human IgG and IgM (Zymed Laboratories, South San Francisco, CA) or avidin horseradish peroxidase (eBioscience, San Diego, CA) as the reporting system. The fluorochrome-conjugated mouse mAb against human IgGs and human CD3 (UCHT1; mouse IgG1), together with rat mAb against mouse IgG2a were commercially available from Becton Dickinson Immunocytometry Systems (San Jose, CA) and Abcam (Cambridge, UK). The anti-CD3 (OKT3; mouse IgG2a) used for T cell activation and the antagonistic anti-CD152 (BNI3; mouse IgG2a) were purchased from eBioscience and Abcam, respectively.

### Preparation of human PBMC

Plasma and buffy coat samples from healthy routine blood donors, screened negative for HIV-1/2, HTLV-I/II, HCV, HBsAg and containing normal levels of alanine transferase (ALT), were obtained from the Tainan and Hualien Blood Centers, Taiwan Blood Services Foundation. Written informed consents were obtained from five repeatedly-healthy regular blood donors after an explanation of the nature, purpose, and potential risks of the study and then 230 ml of whole blood was used for the purpose of site-directed *in vitro *immunization. PBMC's were isolated by density centrifugation on Ficoll-Paque (GE Healthcare Bio-Sciences, Uppsala, Sweden) as described elsewhere.

### Magnetic cell purification and depletion

PBMC's were magnetically labeled with CD45RO MACS^® ^microbeads (Miltenyi Biotec, Bergisch Gladbach, Germany) then separated by a VarioMACS™ (Miltenyi) instrument according to the manufacturer's instructions. The purified CD45RO^+ ^T cells were cultured at a density of 2 × 10^6 ^cells/ml in the culture medium supplemented with 50 μM 2-mercaptoethanol and 10 μg/ml pokeweed mitogen (PWM; Sigma, St. Louis, MO). After 24 h, cells were removed by 400 × g centrifugation to collect CD45RO^+ ^T cell replacing factor. Removal of cytotoxic cell populations, which inhibit *in vitro *immunization [23], was similarly performed by using colloidal super-paramagnetic microbeads conjugated to monoclonal anti-human CD8 and anti-CD56 antibodies (Miltenyi). Removal of IL-10-producing cells was achieved by using rat anti-human IL-10 (SouthernBiotech, Birmingham, AL) and goat anti-rat IgG microbeads (Miltenyi).

### Site-directed *in vitro *immunization

Cytotoxic cell-depleted PBMCs were immunized *in vitro *based on a previously described two-step principle [6]. Primary immunization was performed by incubating the cells for 6 days in a medium containing 10 nM of the heterotopic peptide Ag (QYIKANSKFIGITELAATYMMGNELTFLDDSICT; Fine Research Biochem, Taoyuan, Taiwan), 50 μM 2-mercaptoethanol, 10% heat-inactivated human serum, 0.05 ng/ml recombinant human (rh) IL-2 (eBioscience), and 25% (v/v) CD45RO^+ ^T cell replacing factor. For secondary immunization, 3 × 10^7 ^primary-immunized cells were mixed with the peptide in a flask that had been immobilized overnight with 5 mg/ml of CD40L (CD154; eBioscience) together with 1 × 10^7 ^QYIKANSKFIGITEL (Fine Research Biochem)-stimulated CD4^+ ^T cells and 5 ng/ml rh IL-15 (eBioscience). The cells were cultured for 3–5 days in a medium supplemented with 5% human serum, 50 mM 2-mercaptoethanol and 10 nM heterotopic peptide Ag. The significance of differences between treated and control cultures was established by using Student's t test. A *P *value of less than 0.05 was considered statistically significant.

### Epstein-Barr virus (EBV) infection, ELISA and somatic cell hybridization

The *in vitro *immunized cells were infected with EBV by virus-containing supernatant derived from the EBV-producing marmoset cell line B95-8 (American Type Culture Collection, ATCC CRL 1612; kindly provided by Dr. L.-F. Sheu, Tri-Service General Hospital, Taipei). The infected cells were seeded at 10^5^/well in 96-well plates together with mytomycin (Kyowa Hakko Kogyo, Tokyo, Japan)-treated PBMC as feeder cells (10^4^/well) for the establishment of lymphoblastoid cells and screened for Abs by ELISA.

Ag-specific ELISA was performed by coating 0.25 μg/ml purified rhCD152-muIg, 0.5 μg/ml monoclonal mouse IgG2a (mIgG2a; Ancell), 1 μg/ml bovine serum albumin (BSA; Sigma) or 1 μg/ml tetanus toxoid (TT; ADImmune, Taichung, Taiwan) onto microtitre plates overnight at 4°C. Culture supernatants were diluted to the desired level in 10 mM sodium phosphate buffer (pH 8.0), containing 0 5 M sodium chloride and 0.1% Tween-20. Coated plates were incubated with diluted culture supernatants, washed, incubated with peroxidase-labeled goat antibodies against human IgG and IgM and developed (15 min) by addition of 100 μl of the chromogenic substrate o-phenylaenediamine (OPD) (Sigma). The reaction was stopped after 30 min by adding 1 M sulphuric acid, and the absorbances were read at 490 nm.

Somatic cell hybridization was generated by electrofusion. Briefly, Ag-specific EBV-infected lymphoblastoid cells were fused with heteromyeloma cells [22] in an isotonic medium (280 mM sorbitol, 0.5 mM magnesium acetate, 0.1 mM calcium acetate and 1 mg/ml BSA; pH6.9–7.1). Cell fusion was induced by high-voltage pulses using a BTX Electro Cell Manipulator ECM 2001 (Harvard Apparatus, Holliston, MA). Ag-specific hybrids were selected and cloned by limiting dilution.

### Epitope mapping

To define the specific epitope of human CD152 recognized by the mAb, we used peptide arrays (Genesis Biotech, Taipei, Taiwan and Fine Research Biochem,) containing *in-situ *synthesized peptides immobilized on special membrane. In brief, 1 μg/mL of protein A (Proteus MIDI kit, Pro-Chem, Littleton, MA)-purified mAb was incubated by shaking in room temperature for 2 h. After washing, the membrane-bound mAb was then visualized by diluted anti-human IgG conjugated with peroxidase (Jackson ImmunoResearch Laboratories, West Grove, PA) and FAST™ DAB (Sigma). The amount of bound mAb was calculated by Image-Pro Plus 4.5 software (Media Cybernetics, Silver Spring, MD) on the scanned images.

### Flow cytometry analyses

The surface expression of the CD152 epitope recognized by the present mAb was analyzed using two-color flow cytometry on mitogen-stimulated PBMC's by a FACSCaliber™ flow cytometer and CellQuest™ software (Becton Dickinson Immunocytometry Systems). 2 × 10^6 ^isolated PBMC's were resuspended in supplemented culture medium and treated with either anti-human CD3 (OKT3; final concentrations in culture 10 μg/ml, eBioscience), concanavalin A (Con A; final concentrations in culture 10 μg/ml, Sigma), 10 μg/ml phytohemagglutinin (PHA; final concentrations in culture 1 μg/ml, GE Healthcare Bio-Sciences), a combination of phorbol 12-myristate acetate (PMA; 50 ng/ml, Sigma) + ionomycin (1 μM, Sigma) or a combination of PHA (1 μg/ml) + PMA (50 ng/ml). Logarithmically amplified fluorescence data were collected on 10,000 CD3^+ ^cells. All flow cytometry staining procedures were performed at 4°C in cytometry buffer. For extracellular detection of CD152, activated cells were first surface stained with the mAb or isotype control at 4°C, followed by anti-human IgG-FITC and anti-CD3-PE (Becton Dickinson) staining.

### RT-PCR assays for deduction of Ab primary structures

The Ab primary structures were deduced by cDNA sequencing from cloned trioma cells. Briefly, poly(A)^+ ^RNA was isolated from by using Dynabeads^® ^mRNA DIRECT™ Kit (Invitrogen, Carlsbad, CA). Purified mRNA was then employed as the reaction template in reverse transcription polymerase chain reactions (RT-PCR). The RT-PCR was carried out with Titan One Tube RT-PCR System™ (Roche Diagnostics Corporation, Indianapolis, IN). PCR primers (1 μM) used to amplify human VH and VL were the HuVH-JH set (forward: 5'-caggt caact taagg gagtc tgg-3' and reverse: 5'-tgaga gacgg tgacc gtggt ccc-3') and the HuVλ set (forward: 5'-tccta tgtgc tgact cagcc acc-3' and reverse: 5'-accta ggacg gtgac cttgg tccc-3'), respectively. The 37 temperature cycles include: one 2-min denature cycle of 94°C; 35 cycles of 3-min denaturation at 94°C, 1-min annealing and extension at 68°C; and a final 10-min extension cycle of 68°C. Single banded PCR fragments were seperated by 2% agarose gel electrophoresis. The DNA fragments were purified from gel by Wizard PCR Preps DNA purification system (Promega, Madison, WI). The purified products were subjected to nucleotide sequencing. Sequences were verified (Molecular Clinical Diagnostic Laboratory, Dr. Chip Biotechnology, Inc., Taipei, Taiwan) and converted to corresponding amino acids.

### Isoelectric point electrophoresis and affinity analyses

The isoelectric point of secreted IgG was examined by Novex IEF Gels (Invitrogen). Desalted and dialyzed protein samples were mixed with IEF sample buffer at 1:1 (v/v) ratio. Electrophoresis was performed at following condition: 100 V for 1 h, 200 V for 1 h and 500 V for 30 mins. After electrophoresis, gels were removed from gel cassette and fixed in 10% trichloroacetic acid for 30 mins. Fixed gel was developed by Coomassie brilliant blue staining or silver staining. The broad-range calibration kit for pI determinations (#17-0471-01, pH 3–10; GE Healthcare Bio-Sciences) was included as the standard. The isoelectric point of interested proteins was calculated by Phoretix 2D Elite software (Nonlinear Dynamics, Durham, NC).

The affinity of the mAb was determined against CD152-muIg with an IAsys optical biosensor (Affinity Sensors, Cambridge, UK) according to the manufacturer's instructions. Briefly, 200 μg/ml dialyzed and diluted CD152-muIg was immobilized on the activated surface of carboxymethyl dextran cuvettes in 10 mM of sodium acetate buffer at pH 3.8. After conditioning with 10 mM HCl, immobilization of 2 mg/mL CD152-muIg resulted in a response of 1100 arc sec. This represents the highest immobilization response for CD152 and gives a ligate binding capacity (R_max_) of 300 arc sec. Serial dilutions of the mAb in PBS, *i.e*. 1.34 × 10^-9 ^M, 6.70 × 10^-9 ^M, 1.34 × 10^-8 ^M, 2.68 × 10^-8 ^M and 5.36 × 10^-8 ^M, were added to the CD152-coated cuvettes (final volume, 50 μl). Affinity constants (Kd) were calculated from these measurements as k_diss_/k_ass _by using the FASTFIT^® ^program provided by the manufacturer.

## Abbreviations

CDR- Complementarity determining region.

CTLA-4- Cytotoxic T lymphocyte antigen-4.

IEF- Isoelectric focusing.

Ig- Immunoglobulin.

Kd- Equilibrium dissociation (affinity) constant.

LeuLeuOMe- L-leucyl-L-leucin methyl ester hydrobromide.

mAb- Monoclonal antibody.

PBMC- Peripheral blood mononuclear cells.

PBS- Phosphate buffered saline (20 mM phosphate buffer, pH 7.2 containing 145 mM NaCl).

VH- The variable regions of Ab heavy chain.

VL- The variable regions of Ab light chain.

## Competing interests

The molecules described in this paper have commercial potential and patent applications for the same are being processed.

## Authors' contributions

LTC designed and carried out site-directed *in vitro *immunization. CC delineated the primary structure of the antibody and performed statistical analyses. HMC directed and accomplished the determination of isoelectric point. SCH participated in most experiments and assisted with peptide design. BCW compared the mAb with the existing BNI3. CHC drafted the manuscript in collaboration with LTC. CHC was also responsible for the ethical issues regarding to human studies. All authors read and approved the final manuscript.

## Supplementary Material

Additional file 1 Specific efficiency of *in vitro *stimulation using peptide antigen. The table provided represents the number of wells with specific Ab production after various *in vitro *manipulations. The data were assessed by a statistical analysis and presented as Figure 1A. Click here for file
