# Supplementary material for: Site-directed in vitro immunization leads to a complete human monoclonal IgG4λ that binds specifically to the CDR2 region of CTLA-4 (CD152) without interfering the engagement of natural ligands
Source: BMC Biotechnol. 2007 Aug 23;7:51. doi: 10.1186/1472-6750-7-51 (PMC2025598; doi:10.1186/1472-6750-7-51)
Supplement: Additional file 1 — Specific efficiency of in vitro stimulation using peptide antigen. The table provided represents the number of wells with specific Ab production after various in vitro manipulations. The data were assessed by a statistical analysis and presented as Figure 1A. [file 1472-6750-7-51-S1.doc]

**Supplementary Table 1. Specific efficiency of *in vitro* stimulation using peptide antigen**

|  | Number of wells with specific antibody production* | | | | | | |
| --- | --- | --- | --- | --- | --- | --- | --- |
| Donor | Primary | | | | | Secondary | |
|  | Nil | LeuLeuOMe | CD8 removal | CD56 removal | CD8 & CD56 removal | Nil | IL-10 removal |
| A | 0/67 | 0/77 | 1/70 | 0/77 | 2/62 | 1/44 | 3/77 |
| B | 0/47 | 0/72 | 0/77 | 0/72 | 1/71 | 0/53 | 1/81 |
| C | 0/52 | 1/54 | 2/76 | 1/54 | 1/54 | 0/44 | 2/94 |
| D | 0/65 | 0/68 | 0/69 | 0/68 | 0/71 | 1/62 | 0/68 |
| E | 0/43 | 2/76 | 1/70 | 2/76 | 0/57 | 0/47 | 2/77 |

| Specific efficiency was defined as: | Number of wells containing specific antibody production |
| --- | --- |
|  | Number of wells containing growing lymphoblastoid cells after EBV activation and in vitro immunization |

Specific efficiency was defined as:

*A well containing lymphoblastoid cells was scored as specific antibody-producing if:

- - - 1. the ELISA OD value against recombinant CD!52 was at least five times as high as the OD value for the negative control;
      2. the reactivity index (RI) was >5, where RI = [OD_CD152_ – OD_medium control against CD152_] / [OD_murine IgG2a_ – OD_medium control against murine IgG2a_].
      3. Results obtained after primary and secondary in vitro immunizations were measured by specific IgM and IgG, respectively.
